# Supplementary material for: Mouse fetal growth restriction through parental and fetal immune gene variation and intercellular communications cascade
Source: Nat Commun. 2022 Jul 29;13:4398. doi: 10.1038/s41467-022-32171-w (PMC9338297; doi:10.1038/s41467-022-32171-w)
Supplement: Supplementary file 1 — Supplementary Information [file 41467_2022_32171_MOESM1_ESM.pdf]

Fetal growth restriction through parental and fetal immune gene variation and intercellular communications cascade

Gurman Kaur[1,2,#], Caroline B. M. Porter[2,#], Orr Ashenberg[2], Jack Lee[3], Samantha J. Riesenfeld[2,4], Matan Hofree[2], Maria Aggelakopoulou[5], Ayshwarya Subramanian[2], Subita Balaram Kuttikkatte[1], Kathrine E. Attfield[5], Christiane A. E. Desel[5,6], Jessica L. Davies[5], Hayley G Evans[5], Inbal Avraham-Davidi[2], Lan T. Nguyen[2], Danielle A. Dionne[2], Anna E. Neumann[7], Lise Torp Jensen[8], Thomas R. Barber[1], Elizabeth Soilleux[9], Mary Carrington[10,11], Gil McVean[12], Orit Rozenblatt-Rosen[2,13], Aviv Regev[2,13,14,15,*], Lars Fugger[1,5,8,*]

Supplementary Information File

Supplementary Figure 1

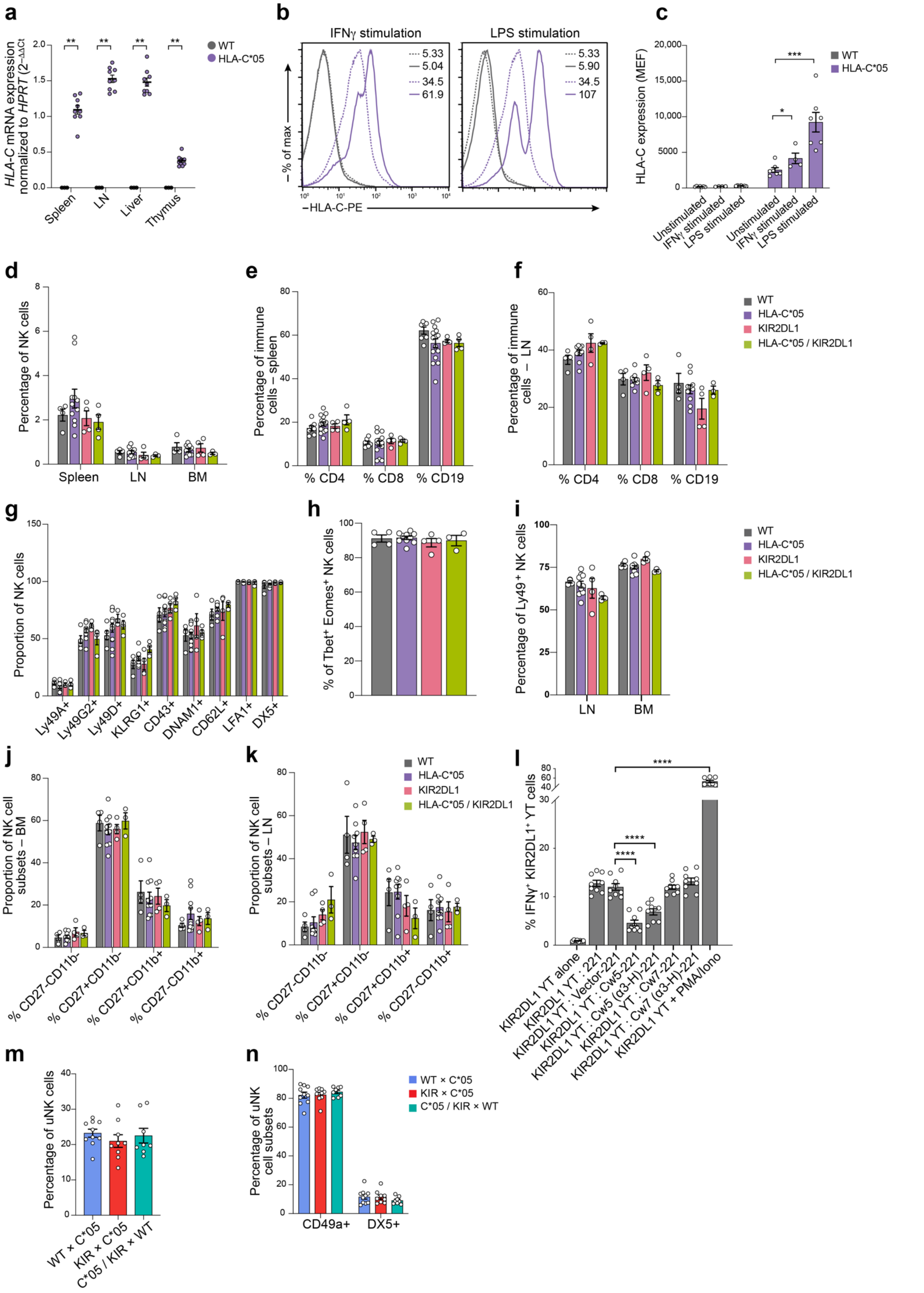

Supplementary Fig. 1. HLA-C*05 and KIR2DL1 transgenic mouse model characterisation. (**a**) *HLA-C*05* expression, normalised to HLA-C*05 spleen. (**b**) Representative HLA-C*05 expression on stimulated (solid lines) and unstimulated (dashed lines) total spleen cells. Numbers denote MFI. (**c**) HLA-C expression (MEF) on total splenocytes. (**d**) NK ($CD3^-TCR^-NKp46^+$) cell percentage in spleen, LN and BM. (**e, f**) $CD3^+CD4^+$, $CD3^+CD8^+$ and $CD19^+$ cell percentage in the spleen (**e**) and LN (**f**). (**g, h**) Splenic NK cells expressing cell surface receptors (**g**), T-bet and Eomes (**h**). (**i**) $Ly49^+$ NK cells (antibody clones 14B11, 4D11) in LN and BM. (**j, k**) CD27 and CD11b staining on gated NK cells from BM (**j**) or LN (**k**). Purple: HLA-C*05, grey: WT, pink: KIR2DL1-expressing, green: HLA-C*05/KIR2DL1 double transgenic. (**l**) Response of KIR2DL1-expressing YT NK cell line co-cultured with 721.221 cells alone, 721.221 cells transduced with lentiviral HLA-C*05, HLA-C*07, HLA-C*05 (α3-H), HLA-C*07 (α3-H), empty-vector or PMA/Calcium Ionomycin. (**m, n**) uNK cells ($CD3^-CD19^-TCR^-CD45^+NKp46^+CD122^+$) (**m**) and $CD49a^+$ or $DX5^+$ uNK cell subsets (**n**) at the maternal-fetal interface at gd9.5 in the transgenic crosses. Mating crosses are written as female x male. Mean ± SEM is shown. n represents biologically independent animals in each group. (**a**) n = 3 (WT), 9 (HLA-C*05). (**c**) n= 7 (Unstim), 4 (IFNγ stim), 7 (LPS stim). (**d-k**) For WT, HLA-C*05, KIR2DL1, and HLA-C*05/KIR2DL1, respectively: (**d**) n = 4, 11, 4, 4 (spleen), n= 4, 10, 4, 3 (LN, BM); (**e**) n = 7, 16, 4, 4; (**f**) n = 4, 10, 4, 3; (**g**) n = 4, 9, 3, 4 (Ly49A), n = 5, 11, 4, 4 (Ly49G2), n = 5, 12, 3, 4 (Ly49D), n=4, 11, 4, 4 (KLRG1, DNAM1, LFA1), n=4, 10, 4, 4 (CD43), n=4, 10, 4, 3 (CD62L, DX5); (**h-j**) n = 4, 10, 4, 3; (**k**) n = 4, 9, 4, 3. (**l**) n = 8 (KIR2DL1-YT alone, PMA/Iono), n=9 otherwise. (**m,n**) n= 10 (WT x C*05), 9 (KIR x C*05), 8 (C*05 / KIR x WT). *p < 0.05, **p < 0.01, ***p < 0.001, ****p < 0.0001, Two-tailed Mann-Whitney U test. Exact p-values are: (**a**) p=0.0091 (all comparisons), (**c**) p=0.0424 (IFNγ stim vs. Unstim), p=0.0006 (LPS stim vs. Unstim). (**l**) p<0.0001 (all comparisons). Source Data are provided as a Source Data file.

Supplementary Figure 2

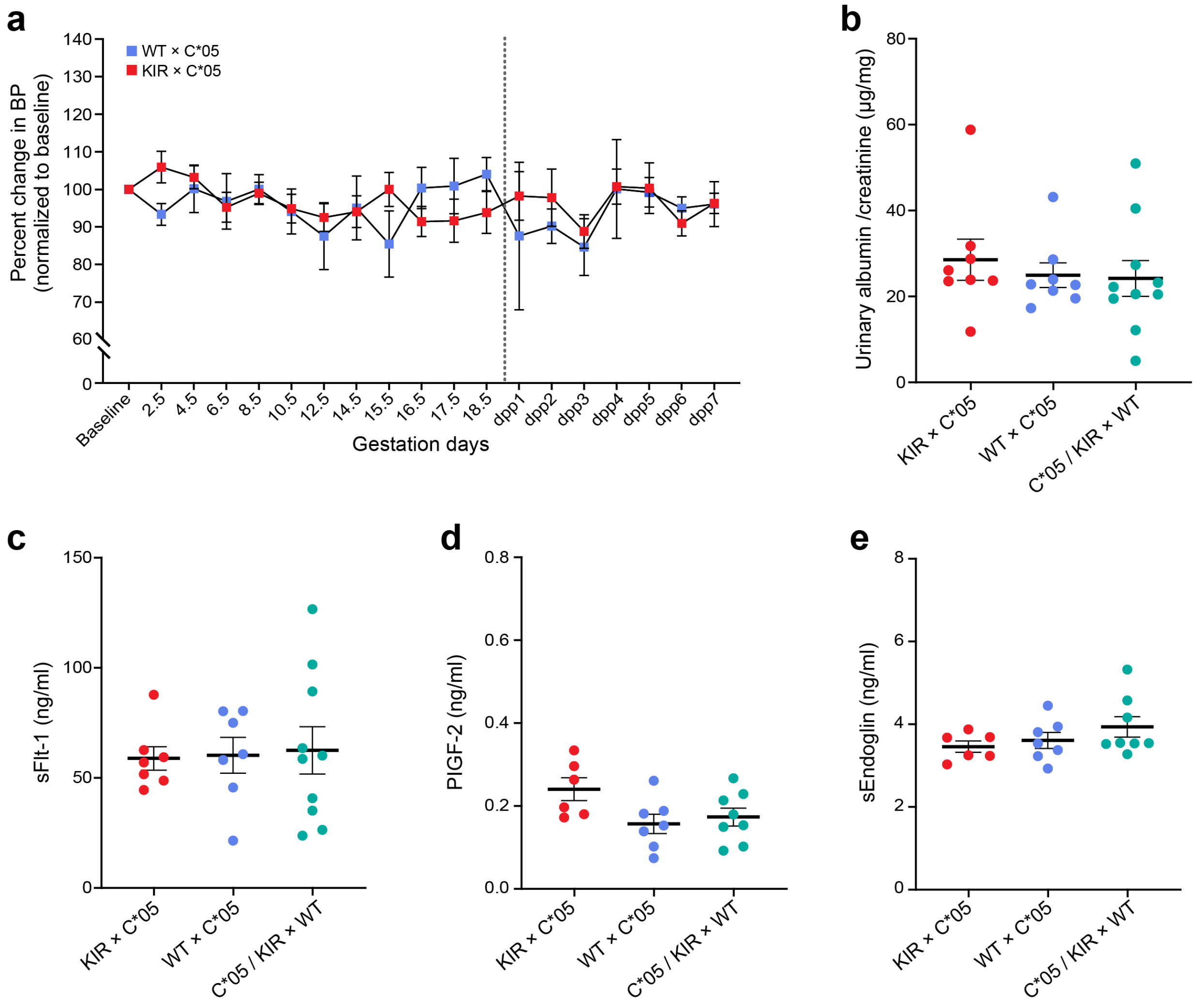

Supplementary Fig. 2. Assessment of blood pressure, urinary protein and plasma proteins. (**a**) Systolic blood pressure measured using non-invasive tail-cuff photoplethysmography during pregnancy and post-partum in the depicted transgenic mating crosses. Grey line indicates birth of the pups. Blood pressure at each time point is shown relative to the baseline for each mouse (measured pre-pregnancy). (**b**) Assessment of protein in urine samples collected from gd18.5 pregnant mice from the different transgenic mating crosses. Urinary albumin/creatinine is shown. (**c, d and e**) Plasma concentrations of sFlt-1 (**c**), PIGF-2 (**d**), sEndoglin (**e**) in pregnant gd18.5 mice from the different transgenic mating crosses. n represents biologically independent animals in each group. (**a**) n= 7 (WT x C*05), 9 (KIR x C*05). Not all mice gave valid blood pressure measurements on every measurement day. Represented data includes observations from a minimum of three mice per group on any measured day. (**b**) n = 8 (WT x C*05), 8 (KIR x C*05) and 10 (C*05 / KIR x WT). (**c**) n = 7 (WT x C*05), 7 (KIR x C*05) and 10 (C*05 / KIR x WT). (**d and e**) n = 7 (WT x C*05), 6 (KIR x C*05) and 8 (C*05 / KIR x WT). Mean ± SEM is shown. Source Data are provided as a Source Data file.

Supplementary Figure 3

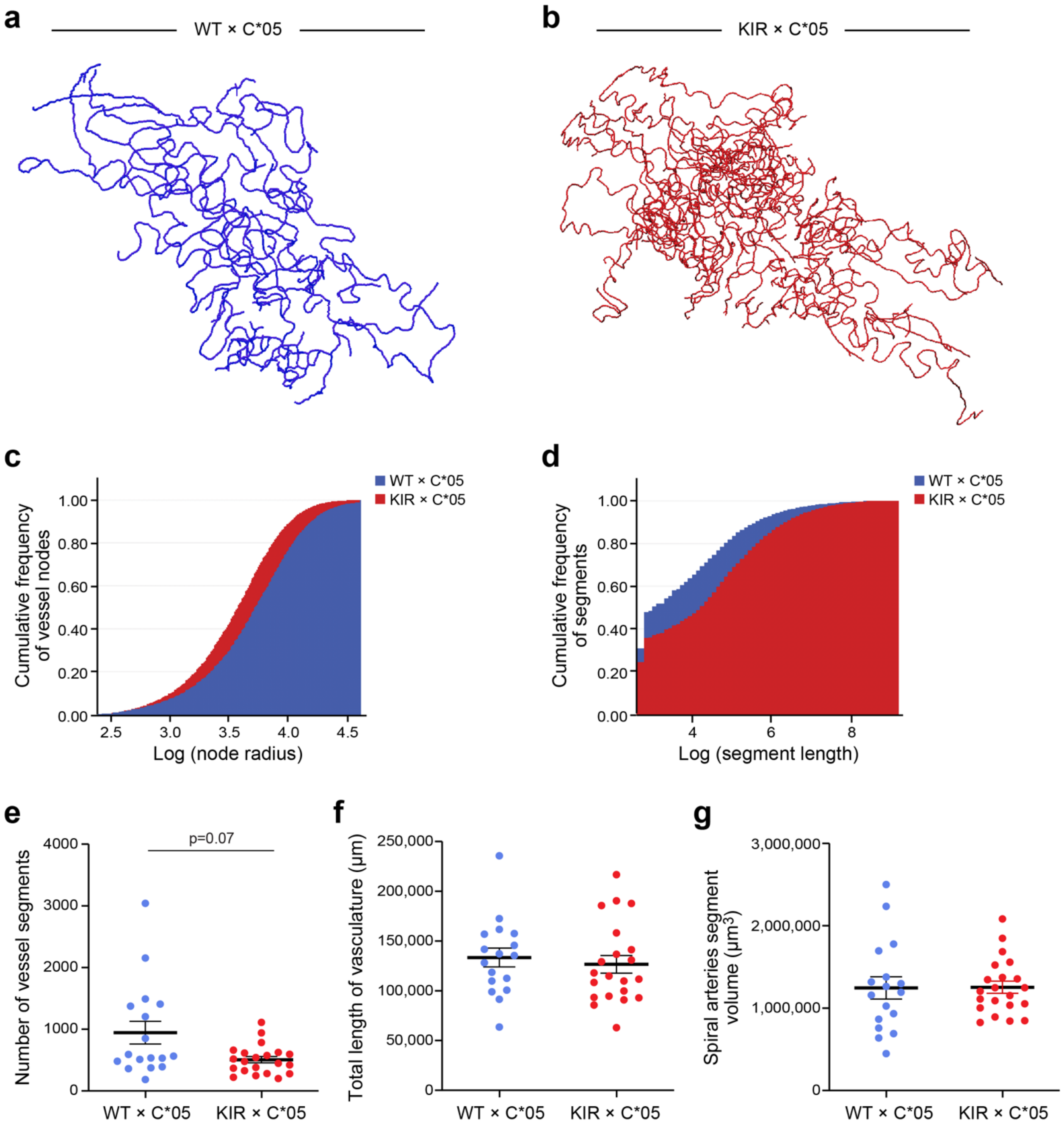

Supplementary Fig. 3. Changes in uterine spiral arteries during gestation. (**a and b**) Representative skeletonised vascular network from the segmentation analysis from the WT x C*05 and KIR x C*05 mating crosses at gd10.5 are shown**.** (**c and d**) Cumulative distributions of vessel radii nodes (**c**) and segment lengths (**d**) from the mating crosses are depicted. (**e**) The number of spiral artery vessel segments are shown. (**f and g**) Total length of vasculature (**f**) and volume (**g**) measured from the spiral artery segmented data from WT x C*05 and KIR x C*05 mating crosses is shown. Data is represented as Mean ± SEM. n represents biologically independent implantation sites from each group. (**c - g**) n = 17 (WT x C*05) and 21 (KIR x C*05). Two-tailed Mann-Whitney U test. Exact p-value was: (**e**) p=0.078. Source Data are provided as a Source Data file.

Supplementary Figure 4

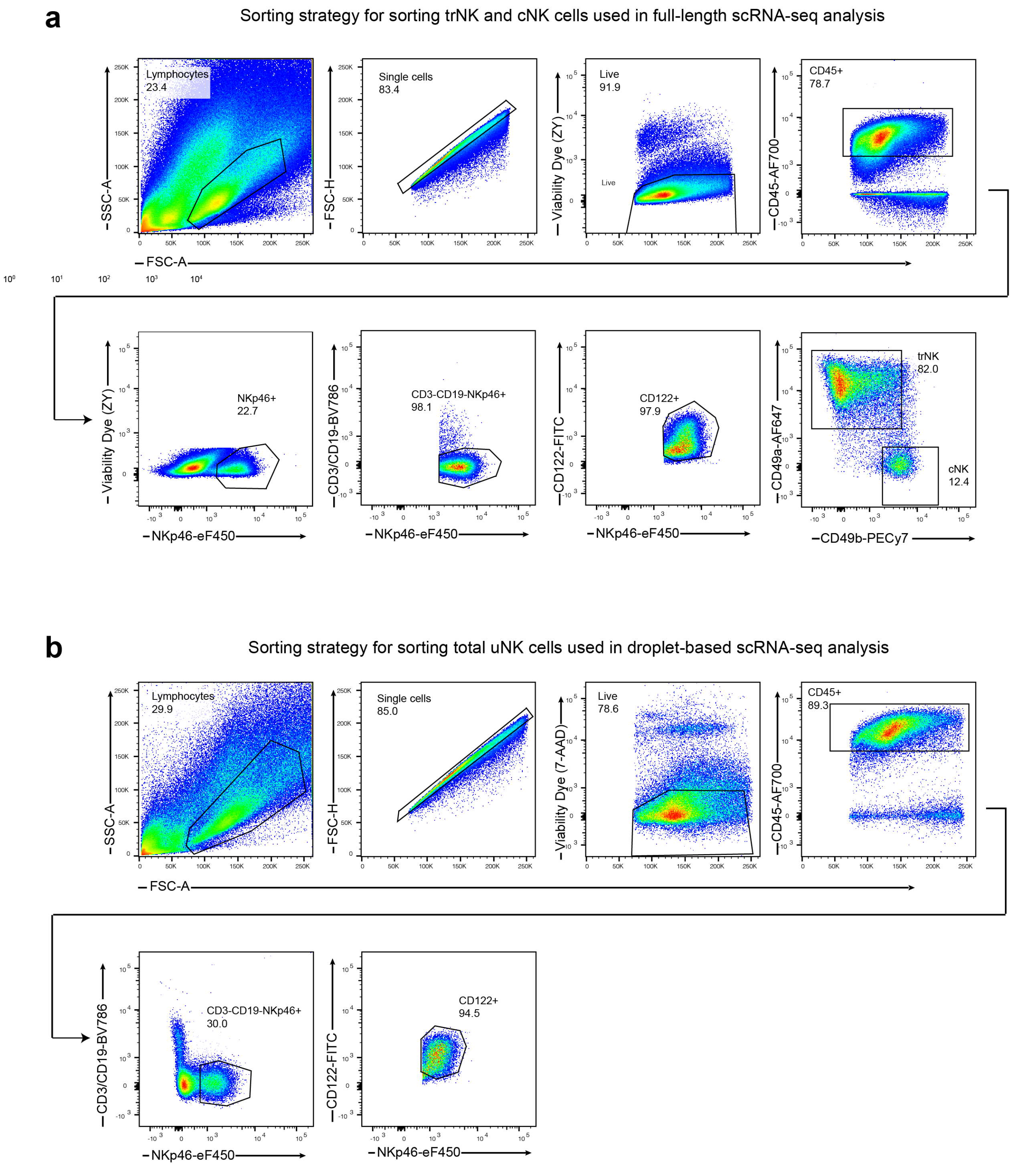

Supplementary Fig. 4. Gating strategy used in FACS. Strategy used for sorting (a) trNK and cNK cells used in full-length scRNA-seq analysis, and (b) total uNK cells used in droplet-based scRNA-seq analyses. Plots are shown from a representative mouse from cells isolated at mid-gestation (gd9.5). Numbers denote percentage of cells within each gate.

Supplementary Figure 5

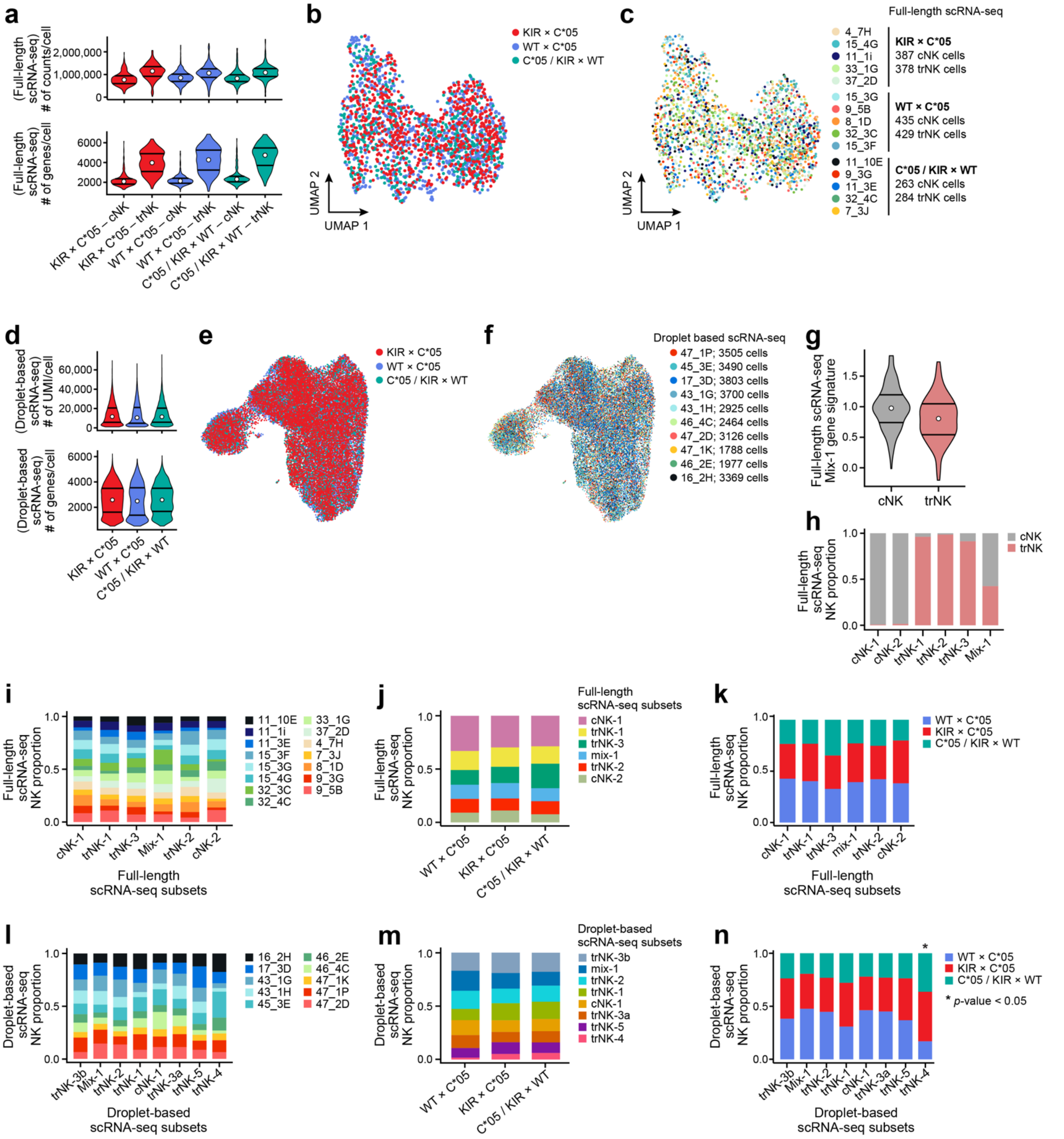

Supplementary Fig. 5. Quality control metrics for full-length and droplet-based scRNA-seq data of sorted NK cells at the mouse maternal-fetal interface. (a) Distribution of the number of counts per cell and the number of genes per cell in each genotype for the full-length sorted NK data. First and third quartiles, and median are shown. Genotypes are displayed along the x axis, and conventional NK cells are indicated by "_cNK" and tissue-resident NK cells are indicated by "_trNK". **(b-c)** UMAP embedding of sorted NK cells from the maternal-fetal interface, analyzed by full-length scRNA-Seq. Colors of the dots correspond to genotype (**b**) or the mouse of origin (**c**). **(d)** Distribution of number of UMI per cell, number of genes per cell in each genotype from the droplet-based scRNA-seq uNK data. First and third quartiles, and median are shown. **(e-f)** UMAP embedding of sorted NK cells analyzed by droplet-based scRNA-seq, with cells colored by genotype (**e**) or mouse of origin (**f**). (**g**) Cells expressing gene signature from the mix-1 cell subset (full-length scRNA-seq data) stratified into cNK or trNK cells based on FACS sorting. (**h**) Proportion of cNK and trNK cells (defined by FACS) in each cell subset for the full-length scRNA-seq NK dataset. (**i-k**) Proportion of composition of clusters depicted in Fig. 5b as per individual mice (**i**) or genotype (**j, k**). (**l-n**) Proportion of composition of clusters depicted in Fig. 5d as per individual mice (**l**) or genotype (**m,n**). $*p < 0.05$, p-values were calculated using a Dirichlet-multinomial regression and represents whether the number of cells belonging to the FGR phenotype (KIR x C*05 mating) are significantly more or less than the two control phenotypes. Exact p-values are (**n**) p=0.011 (trNK-4; KIR x C*05 vs. WT x C*05), p=0.0273 (trNK-4; KIR x C*05 vs C*05/KIR x WT). In all plots, cells from all three genotypes (WT x C*05, KIR x C*05, C*05/KIR x WT) are included. For full-length scRNA-seq, n=5 mice for all three mating groups, k=2176 cells (trNK = 1091 and cNK = 1085). For droplet-based scRNA-seq, n=4 (WT x C*05) and 3 (KIR x C*05 and C*05/KIR x WT), k=30,147 uNK cells. Each mouse represents cells pooled from the maternal-fetal interface of multiple implantation sites within the same litter.

Supplementary Figure 6

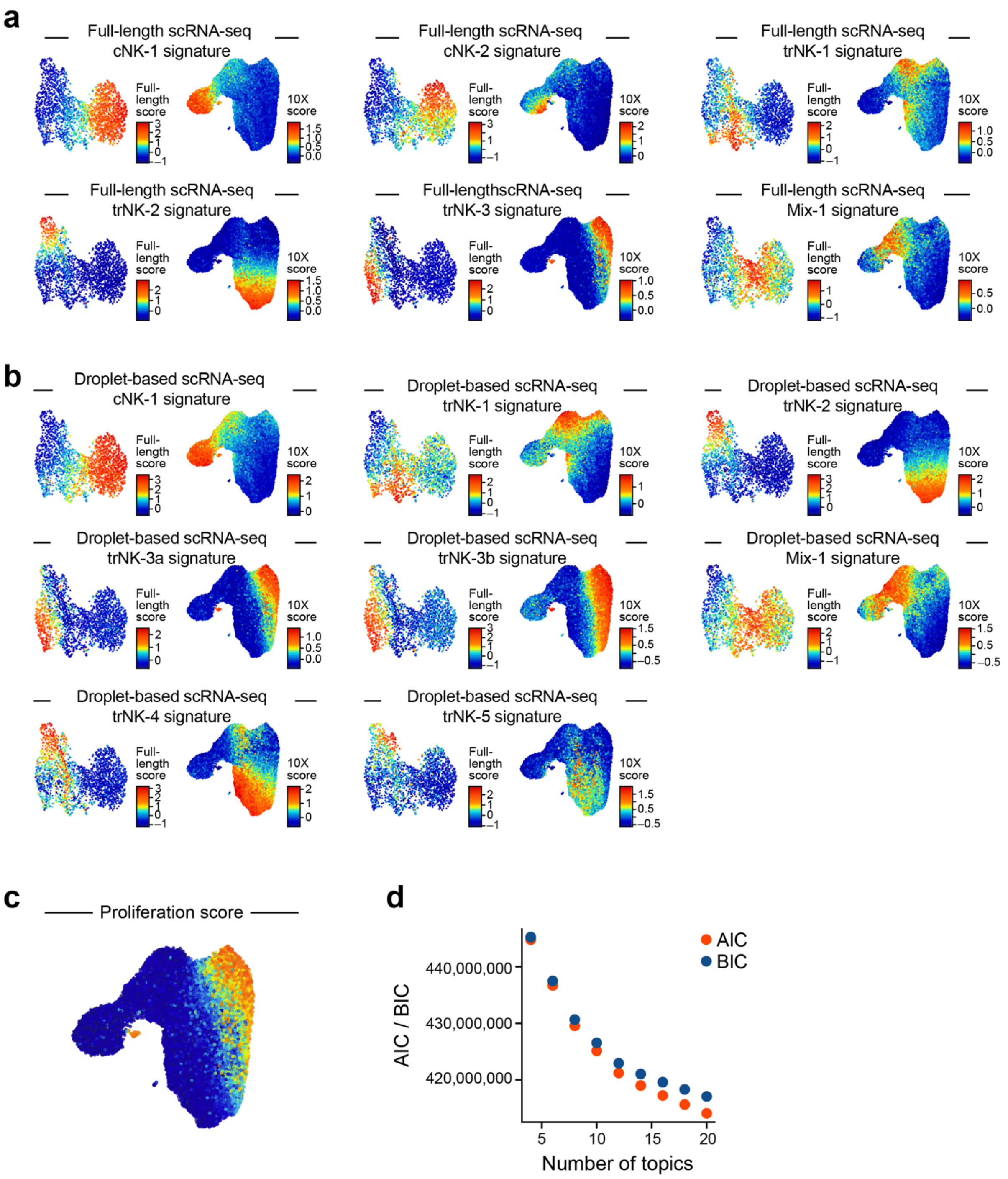

Supplementary Fig. 6. Comparison of NK subtype signatures between the full-length and droplet-based scRNA-seq datasets. (a) NK subtype (cluster) signatures from full-length scRNA-Seq data, plotted on UMAP embeddings for both full-length and droplet-based scRNA-seq data sets to highlight consistency in observed heterogeneity between the two data sets. Red indicates a cell has a high signature score and blue indicates the cell has a low signature score. **(b)** NK subtype (cluster) signatures from the droplet-based scRNA-seq data set, plotted on UMAP embeddings for both full-length and droplet-based scRNA-seq data sets (the reverse of **b**). **(c)** UMAP embedding of droplet-based scRNA-seq NK data, colored by proliferation score. **(d)** Akaike information criterion (AIC) and Bayesian information criterion (BIC) for iterations of topic modeling where k was set to 4:20 in increments of 2; this information was used to help select the value of k (number of topics) to use for our final topic modeling analysis. In all plots, cells from all three genotypes (WT x C*05, KIR x C*05, C*05/KIR x WT) are included. For full-length scRNA-seq, n=5 mice for all three mating groups, k=2176 cells (trNK = 1091 and cNK = 1085). For droplet-based scRNA-seq, n=4 (WT x C*05) and 3 (KIR x C*05 and C*05/KIR x WT), k=30,147 uNK cells. Each mouse represents cells pooled from the maternal-fetal interface of multiple implantation sites within the same litter.

Supplementary Figure 7

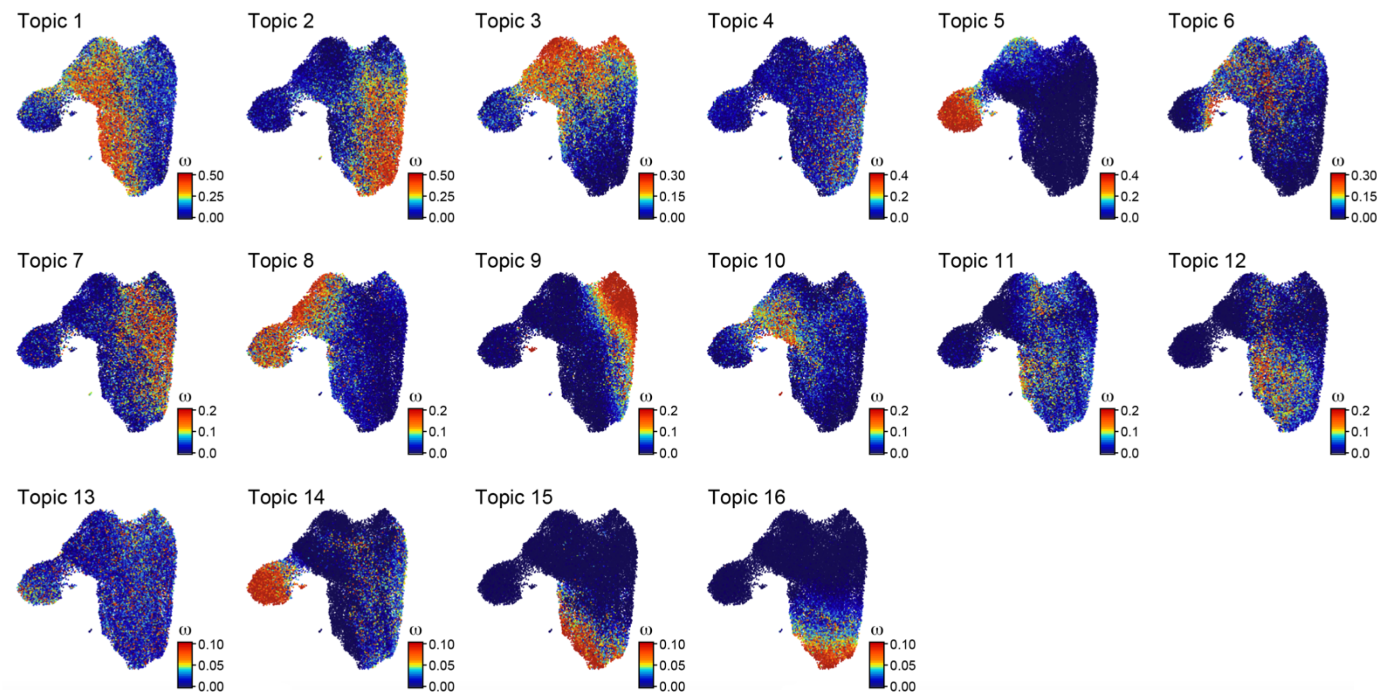

Supplementary Fig. 7. Topic modeling of droplet-based scRNA-seq NK cell data from the maternal-fetal interface. Topics from topic modeling analysis depicting all 16 topics. UMAP embedding colored by the cell weight for that topic – bright red indicates the cell is high in the topic and dark blue indicates the cell is low in the topic. In all plots, cells from all three genotypes (WT x C*05, KIR x C*05, C*05/KIR x WT) are included. n=4 (WT x C*05) and 3 (KIR x C*05 and C*05/KIR x WT), k=30,147 uNK cells. Each mouse represents cells pooled from the maternal-fetal interface of multiple implantation sites within the same litter.

Supplementary Figure 8

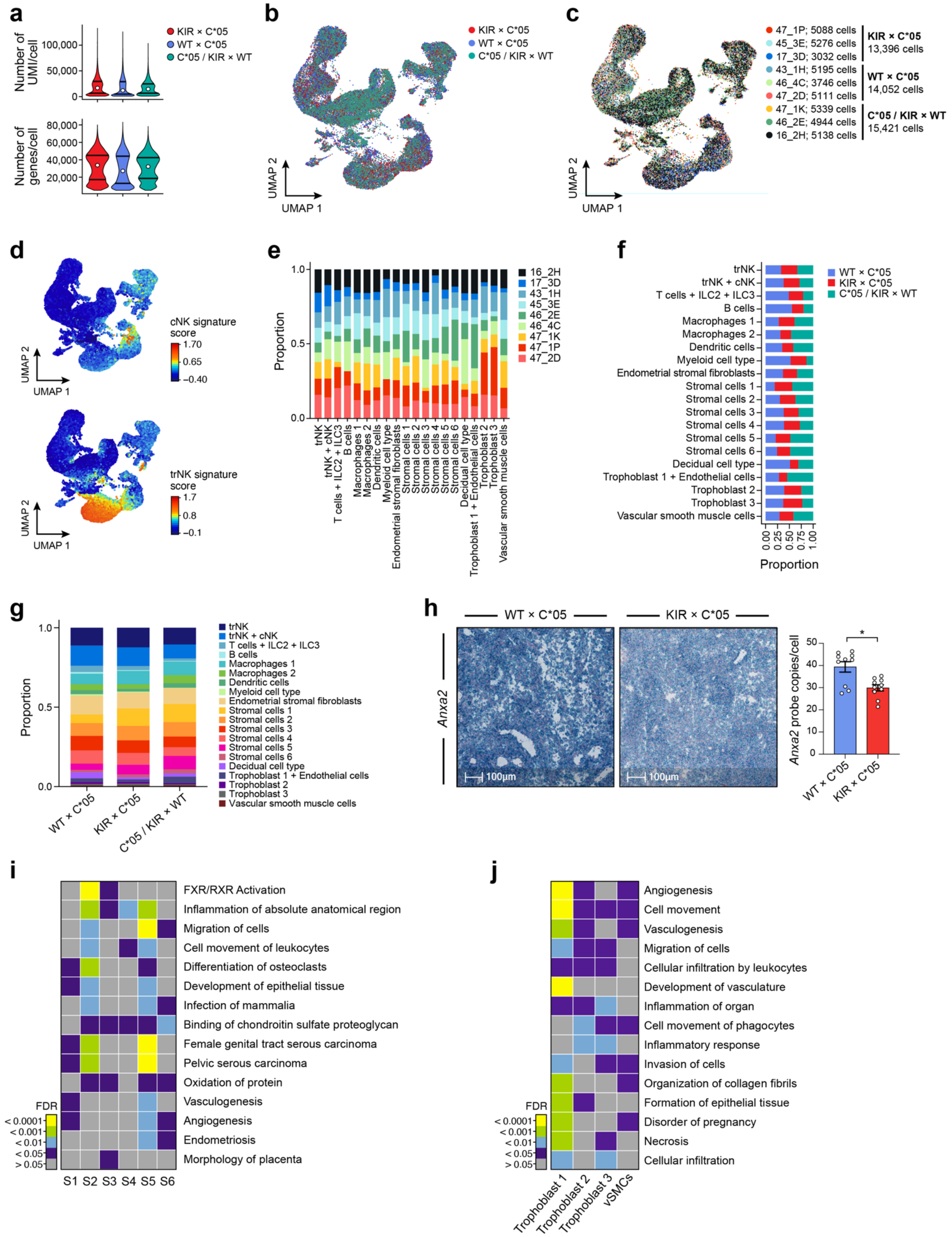

Supplementary Fig. 8. Quality control metrics for droplet-based scRNA-seq data of unsorted cells at the mouse maternal-fetal interface and differentially expressed pathways. (a) Distribution of the number of UMI per cell and the number of genes per cell in each genotype, for droplet-based scRNA-seq data from unsorted cells. **(b-c)** UMAP embedding of unsorted cells analysed by droplet-based scRNA-seq colored by genotype (**b**) or mouse of origin (**c**). **(d)** UMAP embedding of droplet-based scRNA-seq data from unsorted cells colored by cNK and trNK signature score. (**e**) Proportion of composition of clusters/cell types depicted in **Fig. 7b** stratified by individual mice (**e**) or genotype (**f,g**). n = 3 mice each from WT x C*05, KIR x C*05 and C*05 / KIR x WT, k=42,869. Each mouse represents cells pooled from the maternal-fetal interface of multiple implantation sites within the same litter. **(h)** Re-validation of *Anxa2* using *in situ* hybridisation (RNAscope) on uterine tissue collected from implantation sites at gd9.5. Representative probe staining on sections from WT x C*05 or KIR x C*05 implantation sites is shown. *Anxa2* staining is seen in teal. Quantification of probe staining depicted as average probe copies per cell within a stained section is shown as Mean ± SEM. n=10 independent tissue sections per group, *p < 0.05, Two-tailed Mann-Whitney U test. Exact p-value is 0.0232. (**i,j**) Comparison of pathways and functions altered in stromal cell types (**i**), Trophoblast and SMCs (**j**) in KIR x C*05 FGR mice compared to the controls, assessed using Ingenuity pathway analysis. Colors of heatmap indicate BH-FDR value of enrichment. Source Data are provided as a Source Data file.
