# Supplementary material for: Mouse fetal growth restriction through parental and fetal immune gene variation and intercellular communications cascade
Source: Nat Commun. 2022 Jul 29;13:4398. doi: 10.1038/s41467-022-32171-w (PMC9338297; doi:10.1038/s41467-022-32171-w)
Supplement: Supplementary file 3 — Description of Additional Supplementary Files [file 41467_2022_32171_MOESM3_ESM.pdf]

Description of Additional Supplementary Files

Supplementary Data 1 – Differentially expressed genes (marker genes) between cNK and trNK cells from the full-length scRNA-seq data, calculated using Mann-Whitney U test.

Supplementary Data 2 – Genes used for assessment of cell proliferation signature score and housekeeping gene expression.

Supplementary Data 3 – Differentially expressed genes (marker genes) for each NK cell subset from the full-length and droplet-based scRNA-seq data, calculated using Mann-Whitney U test.

Supplementary Data 4 – Top 50 genes from each topic from the topic modelling with latent Dirichlet allocations analysis. Cell weights ('omega') and gene weights ('theta') for each topic are also provided.

Supplementary Data 5 – Differentially expressed genes for each NK cell subset (from the full-length and droplet-based scRNA-seq data) between each mating group i.e. FGR, CTR1 and CTR2, calculated using pseudobulk differential gene expression analysis.

Supplementary Data 6 – Differentially expressed genes (marker genes) for each cell subset from the droplet-based scRNA-seq unsorted cells data.

Supplementary Data 7 – Differentially expressed genes for each subset from the unsorted cells droplet-based scRNA-seq data, between each mating group i.e. FGR, CTR1 and CTR2, calculated using pseudobulk differential gene expression analysis.

Supplementary Data 8 – Analysis of pathways and functions (using Ingenuity Pathway Analysis) performed on differentially expressed genes from selected cell subsets in the droplet-based unsorted cells scRNA-seq data.

Supplementary Data 9 – Cell-cell interaction predictions, calculated from CellPhoneDB for control and FGR mating groups.

Supplementary Data 10 – Details of all oligonucleotides used in the study.

Supplementary Data 11 – Details of all antibodies used in the study.

Supplementary Data 12 – Differentially expressed genes for each NK cell subset (from the full-length and droplet-based scRNA-seq data) between each mating group i.e. FGR, CTR1 and CTR2, calculated using mixed-effect model poisson regression model.

Supplementary Data 13 – Differentially expressed genes for each subset from the unsorted cells droplet-based scRNA-seq data, between each mating group i.e. FGR, CTR1 and CTR2, calculated using mixed-effect model poisson regression model.
