# Supplementary material for: Mouse fetal growth restriction through parental and fetal immune gene variation and intercellular communications cascade
Source: Nat Commun. 2022 Jul 29;13:4398. doi: 10.1038/s41467-022-32171-w (PMC9338297; doi:10.1038/s41467-022-32171-w)
Supplement: Supplementary file 17 — Reporting Summary [file 41467_2022_32171_MOESM17_ESM.pdf]

nature portfolio

Corresponding author(s): Lars Fugger and Aviv Regev

Last updated by author(s): 18th May 2022

Reporting Summary

Nature Portfolio wishes to improve the reproducibility of the work that we publish. This form provides structure for consistency and transparency in reporting. For further information on Nature Portfolio policies, see our Editorial Policies and the Editorial Policy Checklist.

Statistics

For all statistical analyses, confirm that the following items are present in the figure legend, table legend, main text, or Methods section.

n/a	Confirmed	
☐	☒	The exact sample size (*n*) for each experimental group/condition, given as a discrete number and unit of measurement
☐	☒	A statement on whether measurements were taken from distinct samples or whether the same sample was measured repeatedly
☐	☒	The statistical test(s) used AND whether they are one- or two-sided<br>*Only common tests should be described solely by name; describe more complex techniques in the Methods section.*
☐	☒	A description of all covariates tested
☐	☒	A description of any assumptions or corrections, such as tests of normality and adjustment for multiple comparisons
☐	☒	A full description of the statistical parameters including central tendency (e.g. means) or other basic estimates (e.g. regression coefficient) AND variation (e.g. standard deviation) or associated estimates of uncertainty (e.g. confidence intervals)
☐	☒	For null hypothesis testing, the test statistic (e.g. *F*, *t*, *r*) with confidence intervals, effect sizes, degrees of freedom and *P* value noted<br>*Give P values as exact values whenever suitable.*
☒	☐	For Bayesian analysis, information on the choice of priors and Markov chain Monte Carlo settings
☒	☐	For hierarchical and complex designs, identification of the appropriate level for tests and full reporting of outcomes
☒	☐	Estimates of effect sizes (e.g. Cohen's *d*, Pearson's *r*), indicating how they were calculated

Our web collection on statistics for biologists contains articles on many of the points above.

Software and code

Policy information about availability of computer code

Data collection	1. LightCycler® 480 Software, Version 1.5 (Roche)<br>2. Gel Doc XR+ Gel Documentation System with Image Lab™ Software version 6 (Bio-rad)<br>3. BP-2000 Blood Pressure Analysis System (Bioseb)<br>4. Micro-CT imaging was performed on Skyscan 1172 (Bruker)<br>5. BD LSRFortessa (BD Biosciences), Fortessa X-20 (BD Biosciences), BD FACS Aria II and BD FACS Aria III (BD Biosciences) using BD FACSDiva software (v8.0), Cyan ADP (Dako) using summit software (v4.3) and Attune acoustic focusing cytometer (Applied Biosystems) using Attune NxT software (v4.2).
Data analysis	1. HALO® Image Analysis Platform (Indica Labs, Albuquerque, NM USA) for analysis of RNAscope data. Multiple HALO® algorithms were utilized for analysis including ISH v3.3.9 (RNA probe identification) and Multiplex IHC v2.1.1 (cytoplasmic and nuclear staining localisation).<br>2. Systolic blood pressure data were analysed with the BP-2000 Analysis Software v2017.01.12 (Bioseb).<br>3. The micro-CT datasets were manually segmented slice by slice using the seg3D v2.4.3 software and the three-dimensional (3D) images were reconstructed using SkyScan Nrecon v1.7.1 (Micro Photonics).<br>4. FlowJo, Version v.10 was used for analysis of flow cytometry data and plotting.<br>5. GraphPad Prism, Version 8 or RStudio v1.1 or newer, was used for data analysis.<br>6. IPA (Ingenuity Pathway Analysis) version 01-13 and EnrichR (http://amp.pharm.mssm.edu/Enrichr/) were used for pathway enrichment.<br>7. bcl2fastq (v.2.17.1.14) was used for demultiplexing raw data to fastq files for full length scRNA-Seq SMART-Seq2 data.<br>8. RSEM (v.1.2.8) was used for aligning to a custom reference (full length scRNA-Seq SMART-Seq2 data). Quality control was assessed and summaries generated using STAR (v.2.5.1b).<br>9. cellranger mkfastq (version 2.1.0, 10x Genomics) was used for demultiplexing raw sequencing data to fastq files (droplet-based scRNA-Seq data).<br>10. cellranger count (version 2.0.1, 10x Genomics) was used for read alignment and generating count matrices (droplet-based scRNA-Seq

data).
11. cellranger mkref (version 2.1.0, 10x Genomics) was used for generating custom reference.
12. Scrublet (v.0.2.1) was used to predict doublets.
13. Seurat (v.2.1) R package was used for scRNA-seq data analysis. Seurat (v.3.2.2) was used for scoring NK cell subsets.
14. DESeq2 (v.1.26) R package was used for differential expression analysis using pseudobulk approach.
15. fdrtools (v.1.2.16) R package was used to calculate FDR based on the empirical null.
16. lme4 R package (v.1.1-26) was used for the mixed-effects Poisson regression model.
17. EnchancedVolcano (v.1.4.0) R package was used for volcano plots of differentially expressed genes.
18. CountClust (v.1.6.1) R package was used for topic modeling.
19. CellPhoneDB v1.1 (https://github.com/Teichlab/cellphonedb) was used as a tool to identify individual significant ligand-receptor interactions between subsets.
20. Cytoscape v3.8.2 was used for visualising cell subset interactions.
21. Flow cytometric samples were acquired on the LSRFortessa (BD Biosciences), Fortessa X-20 (BD Biosciences) using BD FACSDiva software (v8.0), Cyan ADP (Dako) using summit software (v4.3) or the Attune acoustic focusing cytometer (Applied Biosystems) using Attune NxT software (v4.2). FACS sorting of samples was performed using FACS Aria II or FACS Aria III (BD Biosciences) using BD FACSDiva software (v8.0).
22. The code used for analysis of the single cell RNA-sequencing data presented in this paper is available on https://github.com/GKaur101/Fetal_Growth_Restriction and on Zenodo at https://doi.org/10.5281/zenodo.6538595. The code used for the vascular segmentation analysis presented in this paper is available on https://github.com/jack-kcl/vascular-processing and on Zenodo at https://doi.org/10.5281/zenodo.6553163.

For manuscripts utilizing custom algorithms or software that are central to the research but not yet described in published literature, software must be made available to editors and reviewers. We strongly encourage code deposition in a community repository (e.g. GitHub). See the Nature Portfolio guidelines for submitting code & software for further information.

Data

Policy information about availability of data

All manuscripts must include a data availability statement. This statement should provide the following information, where applicable:
- Accession codes, unique identifiers, or web links for publicly available datasets
- A description of any restrictions on data availability
- For clinical datasets or third party data, please ensure that the statement adheres to our policy

The raw and processed gene expression data in this study have been deposited in the Gene Expression Omnibus database under accession code GSE202983 (https://www.ncbi.nlm.nih.gov/geo/query/acc.cgi?acc=GSE202983).
The processed gene expression data are also available on the Single Cell Portal (https://singlecell.broadinstitute.org/single_cell/study/SCP1312/). All remaining data are provided in the Supplementary Data files, and source data for each figure are available as source data files.

Field-specific reporting

Please select the one below that is the best fit for your research. If you are not sure, read the appropriate sections before making your selection.

☒ Life sciences ☐ Behavioural & social sciences ☐ Ecological, evolutionary & environmental sciences

For a reference copy of the document with all sections, see nature.com/documents/nr-reporting-summary-flat.pdf

Life sciences study design

All studies must disclose on these points even when the disclosure is negative.

Sample size

1. For expression and functional characterisation experiments of transgenic mice, between 3-12 mice per group were used (details in figure legends). These numbers were sufficient to capture any variation and expression changes for the measurement tested. No statistical method was used to pre-determine sample size, and the sample sizes are chosen because they provide sufficient confidence to assess the experimental results.
2. For assessment of blood pressure measurements, 7-9 independent pregnant mice were used per group. This was based on the initial baseline blood pressure measurement experiments that showed that not all mice provide valid blood pressure measurements on every measurement day. Therefore, the maximum number of available mice of the right genotype were used for baseline blood pressure measurements respecting the guidelines of animal welfare. These mice were then set-up in timed-mating experiments and all mice that were detected plugged and pregnant were included in the assessment of blood pressure through the course of gestation and post-partum. These numbers allowed us to have a minimum of 3 independent mice per group on any given measurement day.
3. For assessment of fetal weight between different genotype crosses, between 8-12 independent litters per group were used. These numbers were chosen to have sufficient fetuses that would take into account the effects of the transgenic genotype mating combination and clustering of observations within a litter, and allow assessment of birth weight centiles. The sample size was chosen to assure significant statistical differences and reproducibility of results based on initial pilot experiments that employed a control and FGR group and had 3 independent litters per group. The maximum number of available mice were used respecting the guidelines of animal welfare, ensuring a minimum of eight independent litters per group.
4. For assessment of uterine spiral artery vasculature, 17-21 independent implantation sites per group were used. These represented implantation sites where the spiral artery network was intact and could be confidently segmented. No statistical method was used to pre-determine sample size. The sample sizes are chosen because they provide sufficient confidence to assess the experimental results, and take into account the effect of the transgenic genotype mating combination, clustering of observations from a specific implantation site and

clustering of observations within a litter from a mouse. For assessments of vascular conductance, the maximum number of implantation sites where assessments of flow inlets and outlets could be made were used in the assessment.
5. scRNA-seq experiments: For full length scRNA-seq NK data, 2176 NK cells (trNK = 1091 cells and cNK = 1085 cells) were analysed. These represented cells from WT x C*05 mating combination (5 independent mice), KIR x C*05 mating combination (5 independent mice) and C*05/KIR x WT mating combination (5 independent mice). For droplet based scRNA-seq NK data, 30,147 uNK cells were analysed. These included cells from the three mating groups (WT x C*05, KIR x C*05 and C*05/KIR x WT) and included 3-4 independent mice from each mating combination. For droplet-based scRNA-seq unsorted cell data, 42,869 cells were analysed. These included cells from the three mating groups (WT x C*05, KIR x C*05 and C*05/KIR x WT) and included 3 independent mice from each mating combination. Each mouse represented cells pooled from the maternal-fetal interface of multiple implantation sites within the same litter. No statistical method was used to pre-determine sample size. The sample sizes are chosen because they provide sufficient confidence to assess the results, reveal the cellular complexity of the maternal-fetal interface as well as capture differences between the different genotypes.

Data exclusions

1. For assessment of blood pressure measurements - inclusion of measurements for each mouse required recording of a minimum of 4 valid readings on each day of measurement. Measurements with less than 4 valid readings on any day were excluded.
2. In assessment of uteroplacental vasculature - only fully perfused networks were used. In assessment of conductance measurements - implantation sites where assessments of flow inlets and outlets could not be made were excluded.
3. For full length scRNA-seq, lower-quality cells were removed if they met any of the following criteria: (a) log10(counts) < 5, (b) number of expressed genes < 1,000 or > 7,000, or (c) average housekeeping gene expression (TPM) > 1. In addition, cells that met any of the following criteria were also removed (to ensure cells meet FACS expectations): (d) Cells with HLA-C*05 expression < 2 in C*05/KIR x WT mating group; (e) Cells with HLA-C*05 expression > 2 in WT x C*05 mating group, (f) Cells with KIR2DL1 expression < 2 in KIR x C*05 and C*05/KIR x WT mating groups; (g) Cells with KIR2DL1 expression > 2 in WT x C*05 mating group; (h) Cells with Cd3e expression > 3; (i) In sorted cNK cells, cNK cells with Itga1 expression > 1. Genes expressed in three or fewer cells were removed.
4. For droplet-based scRNA-seq, low quality cells were removed by filtering any cells with either (a) less than 500 genes, (b) less than 1000 UMIs, or (c) more than 10% of UMIs mapped to mitochondrial genes. Genes expressed in three or fewer cells were removed.
5. We used Scrublet, with expected_doublet_rate=0.06, to predict doublets. In the NK cell sorted droplet-based data we removed one cluster with high doublet probability as well as one cluster of non-NK cells, and then re-analyzed the remaining data. In the unsorted droplet-based data we excluded from visualization and analysis cells from two clusters with high doublet scores, but did not reanalyze the data following exclusion.

Replication

Depending on the experiment type, each biological sample represented an independent mouse, an independent sample from pooled implantation sites from a pregnant mouse or an independent implantation site at a specific day of gestation. All protocols were tested on multiple different biological samples (biological replicates) and the protocol performance was consistent between the different samples tested. All reported experiments were performed independently. Technical replicates are not used for determining any statistics.

Randomization

Allocation of samples to the different experimental groups was based on mouse genotyping results. As part of the experimental design, biological samples from different genotypes were tested on any experimental day (where feasible) in order to avoid any technical confounders. In addition, all biological samples from different experimental groups were subject to the same protocols and treatment.

Blinding

As allocation of samples to the different experimental groups required assessment of mouse genotyping results - blinding was not possible. However, where possible, samples from different experimental groups were processed on each experimental day. All samples were subject to same treatment and protocol.

Reporting for specific materials, systems and methods

We require information from authors about some types of materials, experimental systems and methods used in many studies. Here, indicate whether each material, system or method listed is relevant to your study. If you are not sure if a list item applies to your research, read the appropriate section before selecting a response.

Materials & experimental systems

n/a	Involved in the study	
☐	☒	Antibodies
☐	☒	Eukaryotic cell lines
☒	☐	Palaeontology and archaeology
☐	☒	Animals and other organisms
☒	☐	Human research participants
☒	☐	Clinical data
☒	☐	Dual use research of concern

Methods

n/a	Involved in the study	
☒	☐	ChIP-seq
☐	☒	Flow cytometry
☒	☐	MRI-based neuroimaging

Antibodies

Antibodies used

1. Alexa Fluor® 700 anti-mouse CD45 Antibody, clone 30-F11 Biolegend Cat# 103128
2. Cytokeratin 7 Antibody (Alexa Fluor® 488), clone RCK105 Santa Cruz Biotechnology Cat# sc-23876 AF488
3. PE-Cy7 Conjugated anti-mouse CD4 (L3T4), clone RM4-5 eBioscience Cat# 25-0042-81
4. Alexa Fluor® 488 anti-mouse CD4 Antibody, clone RM4-5 Biolegend Cat# 100529
5. PE anti-mouse CD4, clone RM4-5 Biolegend Cat# 100512
6. PE/Cy7 anti-mouse CD326 (Ep-CAM), clone G8.8 Biolegend Cat# 118215
7. Purified Rat Anti-Mouse CD16/CD32 (Mouse BD Fc Block™), clone 2.4G2 BD Biosciences Cat# 553142
8. R-PE conjugate anti-human HLA-BC Thermo Fisher Scientific Cat# (custom-conjugation from Cat#16-5935-82)

9. FITC anti-mouse CD3epsilon, clone 145-2C11 Biolegend Cat# 100306
10. FITC anti-mouse CD4, clone GK1.5 Biolegend Cat# 100406
11. Anti-CD27 FITC, clone LG.7F9 eBioscience Cat# 11-0271-82
12. Anti-mouse Ly-49C/I/F/H FITC, clone 14B11 eBioscience Cat# 11-5991-85
13. Anti-mouse Ly-49G2 FITC, clone eBio4D11 eBioscience Cat# 11-5781-82
14. Anti-mouse KLRG1 FITC, clone 2F1 eBioscience Cat# 11-5893-82
15. FITC anti-mouse CD3e, clone 145-2C11 BD Biosciences Cat# 553062
16. Anti-mouse CD11c FITC, clone N418 eBioscience Cat# 11-0114-85
17. Anti-mouse CD62L FITC, clone MEL-14 eBioscience Cat# 11-0621-85
18. FITC conjugated anti-mouse MHC Class II , clone M5/114.15.2 eBioscience Cat# 11-5321-85
19. PE anti-mouse H-2Kb, clone AF6-88.5 Biolegend Cat# 116508
20. Anti- Human/Mouse T-bet PE, clone eBio4B10 eBioscience Cat# 12-5825-80
21. PE anti-human CD158, clone HP-MA4 Biolegend Cat# 339506
22. PE Rat Anti-mouse CD4, clone RM4-5 BD Biosciences Cat# 553049
23. Anti-mouse CD11b APC, clone M1/70 eBioscience Cat# 17-0112-82
24. Anti-mouse NKp46 CD335 eFluor 660, clone 29A1.4 eBioscience Cat# 50-3351-82
25. Anti-mouse CD226 APC, clone 10E5 eBioscience Cat# 17-2261-80
26. Anti-mouse EOMES eFluor 660, clone Dan11mag eBioscience Cat# 50-4875-80
27. Anti-human HLA-BC APC, clone B1.23.2 eBioscience Cat# 17-5935-42
28. Anti-mouse CD19 APC, clone eBio1D3 eBioscience Cat# 17-0193-82
29. CD11b Monoclonal Antibody APC-eFluor 780, clone M1/70 eBioscience Cat# 47-0112-82
30. Anti-mouse CD19 APC-eFluor 780, clone eBio1D3 eBioscience Cat# 47-0193-82
31. Anti-mouse CD3e APC-eFluor 780, clone 145-2C11 eBioscience Cat# 47-0031-82
32. Anti-mouse TCRβ APC-eFluor 780, clone H57-597 eBioscience Cat# 47-5961-82
33. Brilliant Violet 605 Anti-mouse CD4, clone RM4-5 Biolegend Cat# 100548
34. Anti-mouse CD49b eFluor 450, clone DX5 eBioscience Cat# 48-5971-82
35. Anti-mouse CD19 eFluor 450, clone eBio1D3(1D3) eBioscience Cat# 48-0193-82
36. V450 Mouse Anti-mouse Ly49 A[B6], clone A1 BD Biosciences Cat# 561204
37. Anti-mouse CD122 eFluor 450, clone TM-b1 eBioscience Cat# 48-1222-82
38. Anti-mouse CD335 eFluor 450, clone 29A1.4 eBioscience Cat# 48-3351-82
39. Anti-mouse CD4 eFluor 450, clone RM4-5 eBioscience Cat# 48-0042-82
40. Anti-mouse CD122 PE-Cyanine7, clone TM-b1 eBioscience Cat# 25-1222-80
41. PE-Cy7 Rat Anti-mouse CD43, clone S7 BD Biosciences Cat# 562866
42. Anti-mouse CD8a PE-Cyanine7, clone 53-6.7 eBioscience Cat# 25-0081-81
43. Anti-mouse CD19 PerCP-Cy5.5, clone eBio1D3 eBioscience Cat# 45-0193-82
44. PerCP/Cy5.5 Anti-mouse TCR β chain, clone H57-597 Biolegend Cat# 109228
45. Anti-mouse TER119 PerCP-Cy5.5, clone TER-119 eBioscience Cat# 45-5921-80
46. PerCP/Cy5.5 Anti-mouse CD3e, clone 145-2C11 Biolegend Cat# 100328
47. Anti-Ly49D-PE-Vio770 mouse, clone 4E5 Miltenyi Biotec Cat# 130-102-147
48. Anti-mouse CD49b PEcy7, clone DX5 eBioscience Cat# 25-5971-82
49. Anti-mouse CD122 FITC, clone TM-b1 eBioscience Cat# 11-1222-82
50. Anti-mouse IFNγ APC, clone XMG1.2 eBioscience Cat# 17-7311-82
51. Anti-mouse NK1.1 Percpcy5.5, clone PK136 eBioscience Cat# 45-5941-82
52. Anti-human HLA-BC PE, clone B1.23.2 eBioscience Cat# 12-5935-42
53. Functional grade anti-Ly49D, clone 4E5 Biolegend Cat# 138302
54. Alexa Fluor® 647 Hamster Anti-Rat/Mouse CD49a, clone Ha31/8 BD Biosciences Cat# 562113
55. Anti-mouse H-2Kb/H2-Db eFluor 450, clone AF6-88.5.5.3 eBioscience Cat# 48-5958-82
56. Alexa Fluor® 647 anti-human HLA-A,B,C, clone W6/32 Biolegend Cat# 311414
57. Functional grade anti-NKp46, clone 29A1.4 eBioscience Cat# 16-3351-81
58. Purified anti-human CD158 (KIR2DL1/S1/S3/S5) Antibody, clone HP-MA4 Biolegend Cat# 339502

Validation

All of the antibodies used below were validated by the manufacturer:
1. Alexa Fluor® 700 anti-mouse CD45 Antibody, Application - FC - Quality tested.
Each lot of this antibody is quality control tested by immunofluorescent staining with flow cytometric analysis. The suggested use of this reagent is ≤ 0.25 µg per 106 cells in 100 µl volume.
2. Cytokeratin 7 Antibody (Alexa Fluor® 488), clone RCK105 Santa Cruz Biotechnology Cat# sc-23876 AF488
Cytokeratin 7 (RCK105) is recommended for detection of Cytokeratin 7 of mouse, rat, human, hamster, canine and porcine origin by Western Blotting (starting dilution 1:100, dilution range 1:100-1:1,000), immunoprecipitation [1-2 µg per 100-500 µg of total protein (1 ml of cell lysate)], immunofluorescence (starting dilution 1:50, dilution range 1:50-1:500), immunohistochemistry (including paraffin-embedded sections) (starting dilution 1:50, dilution range 1:50-1:500) and flow cytometry (1 µg per 1 x 106 cells).
3. PE-Cy7 Conjugated anti-mouse CD4 (L3T4), clone RM4-5 eBioscience Cat# 25-0042-81
This RM4-5 antibody has been tested by flow cytometric analysis of mouse thymocytes and splenocytes.
4. Alexa Fluor® 488 anti-mouse CD4 Antibody, clone RM4-5 Biolegend Cat# 100529
Each lot of this antibody is quality control tested by immunofluorescent staining with flow cytometric analysis.
5. PE anti-mouse CD4, clone RM4-5 Biolegend Cat# 100512
Each lot of this antibody is quality control tested by immunofluorescent staining with flow cytometric analysis.
6. PE/Cy7 anti-mouse CD326 (Ep-CAM), clone G8.8 Biolegend Cat# 118215
Each lot of this antibody is quality control tested by immunofluorescent staining with flow cytometric analysis.
7. Purified Rat Anti-Mouse CD16/CD32 (Mouse BD Fc Block™), clone 2.4G2 BD Biosciences Cat# 553142

This antibody is routinely tested by flow cytometric analysis.
8. R-PE conjugate anti-human HLA-BC Thermo Fisher Scientific Cat# (custom-made)
HLA-BC Monoclonal Antibody (B1.23.2), Functional grade, Catalog # 16-5935-82 was conjugated to R-PE.
This B1.23.2 antibody has been tested by flow cytometric analysis of normal human peripheral blood cells.
9. FITC anti-mouse CD3epsilon, clone 145-2C11 Biolegend Cat# 100306
Each lot of this antibody is quality control tested by immunofluorescent staining with flow cytometric analysis.
10. FITC anti-mouse CD4, clone GK1.5 Biolegend Cat# 100406
Each lot of this antibody is quality control tested by immunofluorescent staining with flow cytometric analysis.
11. Anti-CD27 FITC, clone LG.7F9 eBioscience Cat# 11-0271-82
The LG.7F9 antibody has been tested by flow cytometric analysis of mouse splenocytes.
12. Anti-mouse Ly-49C/I/F/H FITC, clone 14B11 eBioscience Cat# 11-5991-85
The 14B11 antibody has been tested by flow cytometric analysis of mouse splenocytes
13. Anti-mouse Ly-49G2 FITC, clone eBio4D11 eBioscience Cat# 11-5781-82
This eBio4D11 (4D11) antibody has been tested by flow cytometric analysis of mouse splenocytes.
14. Anti-mouse KLRG1 FITC, clone 2F1 eBioscience Cat# 11-5893-82
This 2F1 antibody has been tested by flow cytometric analysis of mouse splenocytes.
15. FITC anti-mouse CD3e, clone 145-2C11 BD Biosciences Cat# 553062
Application - Flow Cytometry - routinely tested
16. Anti-mouse CD11c FITC, clone N418 eBioscience Cat# 11-0114-85
The N418 antibody has been tested by flow cytometric analysis of mouse splenocytes
17. Anti-mouse CD62L FITC, clone MEL-14 eBioscience Cat# 11-0621-85
The MEL-14 antibody has been tested by flow cytometric analysis of mouse splenocytes.
18. FITC conjugated anti-mouse MHC Class II , clone M5/114.15.2 eBioscience Cat# 11-5321-85
The M5/114.15.2 antibody has been tested by flow cytometric analysis of mouse splenocytes
19. PE anti-mouse H-2Kb, clone AF6-88.5 Biolegend Cat# 116508
Each lot of this antibody is quality control tested by immunofluorescent staining with flow cytometric analysis.
20. Anti- Human/Mouse T-bet PE, clone eBio4B10 eBioscience Cat# 12-5825-80
The eBio4B10 monoclonal antibody reacts with mouse, rhesus moneky and human T-bet. This eBio4B10 antibody has been reported for use in intracellular staining followed by flow cytometric analysis.
21. PE anti-human CD158, clone HP-MA4 Biolegend Cat# 339506
Each lot of this antibody is quality control tested by immunofluorescent staining with flow cytometric analysis.
22. PE Rat Anti-mouse CD4, clone RM4-5 BD Biosciences Cat# 553049
This antibody is routinely tested by flow cytometric analysis
23. Anti-mouse CD11b APC, clone M1/70 eBioscience Cat# 17-0112-82
The M1/70 antibody has been tested by flow cytometric analysis of mouse splenocytes.
24. Anti-mouse NKp46 CD335 eFluor 660, clone 29A1.4 eBioscience Cat# 50-3351-82
This 29A1.4 antibody has been tested by flow cytometric analysis of mouse splenocytes.
25. Anti-mouse CD226 APC, clone 10E5 eBioscience Cat# 17-2261-80
This 10E5 antibody has been tested by flow cytometric analysis of stimulated mouse splenocytes.
26. Anti-mouse EOMES eFluor 660, clone Dan11mag eBioscience Cat# 50-4875-80
This Dan11mag antibody has been tested by intracellular staining and flow cytometric analysis of mouse splenocytes using the Foxp3/Transcription Factor Buffer Set (cat. 00-5523) and protocol.
27. Anti-human HLA-BC APC, clone B1.23.2 eBioscience Cat# 17-5935-42
This B1.23.2 antibody has been pre-titrated and tested by flow cytometric analysis of normal human peripheral blood cells.
28. Anti-mouse CD19 APC, clone eBio1D3 eBioscience Cat# 17-0193-82
This eBio1D3 (1D3) antibody has been tested by flow cytometric analysis of mouse splenocytes.
29. CD11b Monoclonal Antibody APC-eFluor 780, clone M1/70 eBioscience Cat# 47-0112-82
This M1/70 antibody has been tested by flow cytometric analysis of mouse bone marrow cells.
30. Anti-mouse CD19 APC-eFluor 780, clone eBio1D3 eBioscience Cat# 47-0193-82
This eBio1D3 (1D3) antibody has been tested by flow cytometric analysis of mouse splenocytes.
31. Anti-mouse CD3e APC-eFluor 780, clone 145-2C11 eBioscience Cat# 47-0031-82
This 145-2C11 antibody has been tested by flow cytometric analysis of mouse splenocytes.
32. Anti-mouse TCRβ APC-eFluor 780, clone H57-597 eBioscience Cat# 47-5961-82
This H57-597 antibody has been tested by flow cytometric analysis of mouse splenocytes.
33. Brilliant Violet 605 Anti-mouse CD4, clone RM4-5 Biolegend Cat# 100548
Each lot of this antibody is quality control tested by immunofluorescent staining with flow cytometric analysis.
34. Anti-mouse CD49b eFluor 450, clone DX5 eBioscience Cat# 48-5971-82
This DX5 antibody has been tested by flow cytometric analysis of mouse splenocytes.
35. Anti-mouse CD19 eFluor 450, clone eBio1D3(1D3) eBioscience Cat# 48-0193-82
This eBio1D3 (1D3) antibody has been tested by flow cytometric analysis of mouse splenocytes.
36. V450 Mouse Anti-mouse Ly49 A[B6], clone A1 BD Biosciences Cat# 561204
Application - Flow cytometry - Routinely Tested
37. Anti-mouse CD122 eFluor 450, clone TM-b1 eBioscience Cat# 48-1222-82
This TM-b1 (TM-beta1) antibody has been tested by flow cytometric analysis of mouse splenocytes.
38. Anti-mouse CD335 eFluor 450, clone 29A1.4 eBioscience Cat# 48-3351-82
This 29A1.4 antibody has been tested by flow cytometric analysis of mouse splenocytes.
39. Anti-mouse CD4 eFluor 450, clone RM4-5 eBioscience Cat# 48-0042-82
This RM4-5 antibody has been tested by flow cytometric analysis of mouse splenocytes.
40. Anti-mouse CD122 PE-Cyanine7, clone TM-b1 eBioscience Cat# 25-1222-80

This TM-b1 (TM-beta1) antibody has been tested by flow cytometric analysis of mouse splenocytes.
41. PE-Cy7 Rat Anti-mouse CD43, clone S7 BD Biosciences Cat# 562866
Application - Flow cytometry - Routinely Tested
42. Anti-mouse CD8a PE-Cyanine7, clone 53-6.7 eBioscience Cat# 25-0081-81
This 53-6.7 antibody has been tested by flow cytometric analysis of mouse thymocytes and splenocytes.
43. Anti-mouse CD19 PerCP-Cy5.5, clone eBio1D3 eBioscience Cat# 45-0193-82
This eBio1D3 (1D3) antibody has been tested by flow cytometric analysis of mouse splenocytes.
44. PerCP/Cy5.5 Anti-mouse TCR β chain, clone H57-597 Biolegend Cat# 109228
Each lot of this antibody is quality control tested by immunofluorescent staining with flow cytometric analysis.
45. Anti-mouse TER119 PerCP-Cy5.5, clone TER-119 eBioscience Cat# 45-5921-80
This TER-119 antibody has been tested by flow cytometric analysis of mouse splenocytes.
46. PerCP/Cy5.5 Anti-mouse CD3e, clone 145-2C11 Biolegend Cat# 100328
Each lot of this antibody is quality control tested by immunofluorescent staining with flow cytometric analysis.
47. Anti-mouse anti-Ly49D-PE-Vio770 , clone 4E5 Miltenyi Biotec Cat# 130-102-147
The antibody is QC tested; Applications - Flow cytometry
48. Anti-mouse CD49b PEcy7, clone DX5 eBioscience Cat# 25-5971-82
This DX5 antibody has been tested by flow cytometric analysis of mouse splenocytes.
49. Anti-mouse CD122 FITC, clone TM-b1 eBioscience Cat# 11-1222-82
This TM-b1 (TM-beta1) antibody has been tested by flow cytometric analysis of mouse splenocytes.
50. Anti-mouse IFNγ APC, clone XMG1.2 eBioscience Cat# 17-7311-82
The APC conjugated XMG1.2 antibody has been tested by intracellular staining and flow cytometric analysis of stimulated mouse splenocytes and can be used at less than or equal to 0.125 µg per test.
51. Anti-mouse NK1.1 Percpcy5.5, clone PK136 eBioscience Cat# 45-5941-82
This PK136 antibody has been tested by flow cytometric analysis of C57Bl/6 mouse splenocytes.
52. Anti-human HLA-BC PE, clone B1.23.2 eBioscience Cat# 12-5935-42
This B1.23.2 antibody has been pre-titrated and tested by flow cytometric analysis of normal human peripheral blood cells.
53. Functional grade anti-Ly49D, clone 4E5 Biolegend Cat# 138302
Each lot of this antibody is quality control tested by immunofluorescent staining with flow cytometric analysis.
54. Alexa Fluor® 647 Hamster Anti-Rat/Mouse CD49a, clone Ha31/8 BD Biosciences Cat# 562113
Applications - Flow cytometry - Routinely tested.
55. Anti-mouse H-2Kb/H2-Db eFluor 450, clone AF6-88.5.5.3 eBioscience Cat# 48-5958-82
This AF6-88.5.5.3 antibody has been tested by flow cytometric analysis of mouse splenocytes.
56. Alexa Fluor® 647 anti-human HLA-A,B,C, clone W6/32 Biolegend Cat# 311414
Each lot of this antibody is quality control tested by immunofluorescent staining with flow cytometric analysis.
57. Functional grade anti-NKp46, clone 29A1.4 eBioscience Cat# 16-3351-81
This 29A1.4 antibody has been tested by flow cytometric analysis of mouse splenocytes.
58. Purified anti-human CD158 (KIR2DL1/S1/S3/S5) Antibody, clone HP-MA4 Biolegend Cat# 339502
Each lot of this antibody is quality control tested by immunofluorescent staining with flow cytometric analysis.

Eukaryotic cell lines

Policy information about cell lines

Cell line source(s)	The B-lymphoblastoid cell line, 721.221 was purchased from the International Histocompatibility Working Group. HEK 293T cells were purchased from American Type Culture Collection (ATCC). YT cells transfected with KIR2DL1 were a gift of Dr. Chiwen Chang, University of Cambridge. The originally source of YT cells is as follows: Yodoi, J. et al. TCGF (IL 2)-receptor inducing factor(s). I. Regulation of IL 2 receptor on a natural killer-like cell line (YT cells). J Immunol 134, 1623-1630 (1985).
Authentication	None of the cell lines were authenticated
Mycoplasma contamination	All cell lines tested negative for mycoplasma contamination
Commonly misidentified lines (See ICLAC register)	No commonly misidentified cell lines were used in the study

Animals and other organisms

Policy information about studies involving animals; ARRIVE guidelines recommended for reporting animal research

Laboratory animals	Species: Mus Musculus. Mice were maintained in a pathogen-free facility in individually ventilated cages in an ambient temperature- and humidity-controlled room with a 12h light/12h dark cycle under standard housing conditions with continuous access to food and water. The HLA-C*05 and KIR2DL1 transgenic mice were generated using targeted insertion into the ROSA26 locus. For targeted insertion at the Gt(ROSA26)Sor locus, a PhiC31 integrase mediated cassette exchange approach was adopted using IDG26.10-3 ES cells, which are a (C57BL/6J x 129S6/SvEvTac) F1 ES cell line harbouring a PGK promoter driven hygromycin selection cassette flanked by PhiC31 attP sites, positioned within intron 1 of Gt(ROSA26)Sor 133. The targeted insertion of the vector into the ROSA26 locus ensures reproducible transgene expression. Correctly integrated ES cell clones were injected into mouse C57BL/6J blastocysts, and the resulting chimeric males were mated to C57BL/6J females and the progeny were screened for germline transgene transmission, normal breeding and appropriate expression of the transgene. The mice were further backcrossed onto C57BL/6J for 4-5 generations. Mice at the same backcrossing generation were used as transgene-negative littermates or controls within each experiment. Male and female mice were used across different experiments. For timed-mating experiments, male and female mice were mated in the afternoon of one day and checked for plugs and separated on the morning of the next day. Detection of the plug was considered as

	gd0.5. In addition, female mice were weighed prior to being set-up in timed-mating experiments, and on every alternate day from gd7.5 onwards in order to confirm pregnancy. The NCR1-iCre mice were a gift from Veronika Sexl. The H2-KbH2-Db double-knockout mice were a gift of Petter Höglund.
Wild animals	The study did not involve wild animals
Field-collected samples	The study did not involve samples collected from the field.
Ethics oversight	All animal experiments were approved by the local Ethical Review Committee at the University of Oxford, and performed under license from the UK home office (project license numbers 30/3386 and P0A53015F) in accordance with the Animals (Scientific Procedures) Act, 1986.

Note that full information on the approval of the study protocol must also be provided in the manuscript.

Flow Cytometry

Plots

Confirm that:

☒ The axis labels state the marker and fluorochrome used (e.g. CD4-FITC).

☒ The axis scales are clearly visible. Include numbers along axes only for bottom left plot of group (a 'group' is an analysis of identical markers).

☒ All plots are contour plots with outliers or pseudocolor plots.

☒ A numerical value for number of cells or percentage (with statistics) is provided.

Methodology

Sample preparation	Staining of mouse cells: Surface staining was done in the presence of anti-CD16/32 antibodies to block FcγRII/III receptors using Mouse Fc blockTM (BD Biosciences). Cells were incubated with the Mouse Fc blockTM for 10 min followed by incubation with fluorochrome-conjugated pre-titrated antibodies for 20 min at 4ºC. Cells were washed in FACS buffer (PBS with 2% Heat-inactivated FCS) and fixed in 1x BD cellFIX (BD Biosciences). For intracellular staining, cells were fixed, permeabilised and stained using the BD Cytofix/Cytoperm kit (BD biosciences) as per manufacturer's instructions. For nuclear staining of transcription factors, cells were stained using the Foxp3 staining buffer set (eBioscience) according to the manufacturer's instructions.
Instrument	Samples were acquired on the LSRFortessa (BD Biosciences), Fortessa X-20 (BD Biosciences), Cyan ADP (Dako) or the Attune acoustic focusing cytometer (ThermoFisher Scientific). In order to ensure comparability, FluoroSpheres (Dako) and SpheroTM Rainbow Calibration beads (BD Biosciences) both with defined MEF (molecules of equivalent fluorochromes) were run for each experiment in addition to the samples and used to calculate normalised MFI values.
Software	Data was collected using the BD FACSDiva (BD Biosciences), Summit (Dako) or the Attune NxT software (ThermoFisher Scientific). Data was analysed using the FlowJo software (FlowJo).
Cell population abundance	The total uNK cells were sorted based on staining of cell surface markers and defined as NCR1+CD3ε-TCRb-CD19-CD45 +CD122+. The cNK and trNK populations were distinguished as CD49b+ and Cd49a+ respectively. Based on the sort gates, the relative abundance of the total uNK cells was enriched from ~ 3- 5 % of the total unsorted cells to ~ 90 -96% of the total cells. Single stained as well as FMO controls were used for setting the gates. The purity of the sorted populations was assessed by running the sorted and stained cells population on the flow cytometer and staining using the CD45 (30-F11), CD3 (145-2C11), CD19 (eBio1D3), TCRb (H57-597), NKp46 (29A1.4), CD122 (TM-b1), CD49a (Ha31/8), CD49b (DX5) along with either 7-AAD (eBioscience) or a Live/Dead viability dye (Biolegend). Typically, the purity of the sorted populations was typically > 90-96%.
Gating strategy	For SMART-Seq2 processing, single cells were FACS sorted into 96 U-well plates into either trNK (NCR1+CD3ε-TCRb-CD19-CD45+CD122+CD49a+) or cNK (NCR1+CD3ε-TCRb-CD19-CD45+CD122+CD49b+) cells. For droplet based scRNA-seq, total uNK cells (NCR1+CD3ε-TCRb-CD19-CD45+CD122+) were sorted.<br><br>The below represents a typical example from one of the cNK and trNK cell sorting experiments used for full-length scRNA-seq SMART-Seq2 set-up: Cells were gated by FSC and SSC (23.9% of events retained), doublets removed using FSC-A and FSC-H (83.6% singlets), live cells identified using Live/Dead viability dye (92.1% of cells retained), the distribution of immune cells quantified using the CD45 antibody (78.9% of cells are CD45+), staining of NKp46+ cells (22% of the cells retained), stained for cells that are negative for CD3 and CD19 and positive for NKp46 (98.1% retained), cells that are positive for CD122 (96.5% cells retained), distribution of cNK and trNK cells qualified using the CD49a and CD49b antibody (Cd49a+ are 84.7% and Cd49b+ are 12%).<br><br>The below represents a typical example from one of the total uNK sorting experiments used for droplet-based scRNA-seq: Cells were gated by FSC and SSC (29.9% of events retained), doublets removed using FSC-A and FSC-H (84.7% singlets), live cells identified using 7-AAD (78.9% of cells retained), the distribution of immune cells quantified using the CD45 antibody (89.1% of cells are CD45+), staining for cells that are negative for CD3 and CD19 and positive for NKp46 (30.1% retained), cells that are positive for CD122 (95.1% cells retained).

☒ Tick this box to confirm that a figure exemplifying the gating strategy is provided in the Supplementary Information.
